# Supplementary material for: A collaborative inference strategy for medical image diagnosis in mobile edge computing environment
Source: PeerJ Comput Sci. 2025 Mar 5;11:e2708. doi: 10.7717/peerj-cs.2708 (PMC11935760; doi:10.7717/peerj-cs.2708)
Supplement: Supplemental Information 2 [file peerj-cs-11-2708-s002.docx]

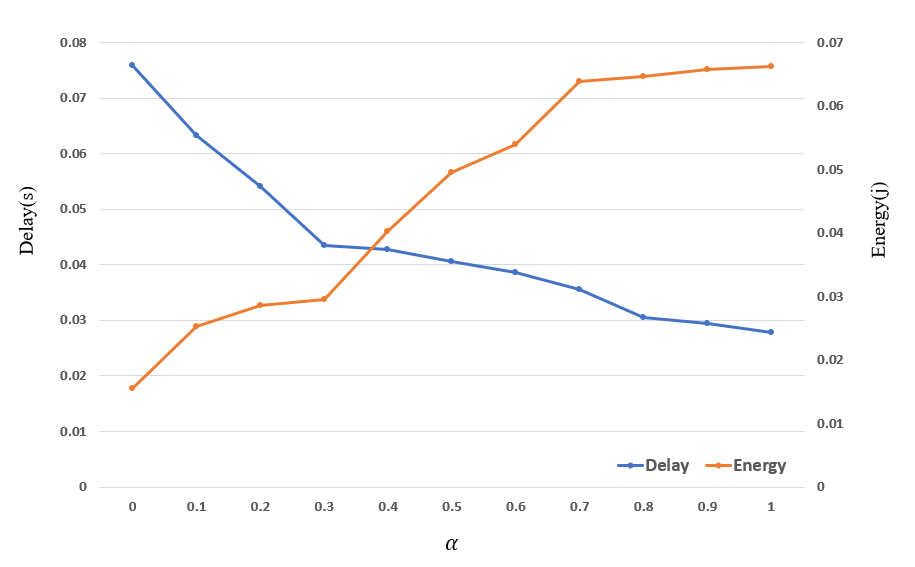


Figure S1: Effect of different α on delay and power consumption


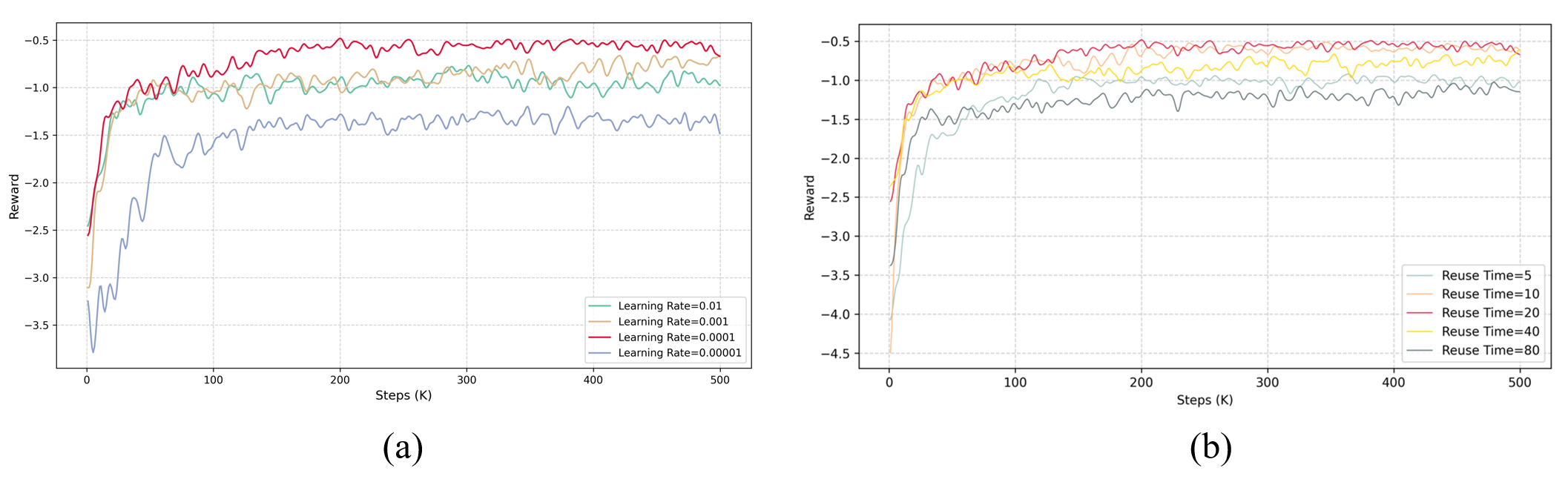


Figure S2. Convergence trend of ResNet18 under different parameter metrics

(a) Convergence trend at different learning rates. (b) Convergence trend for different reuse times


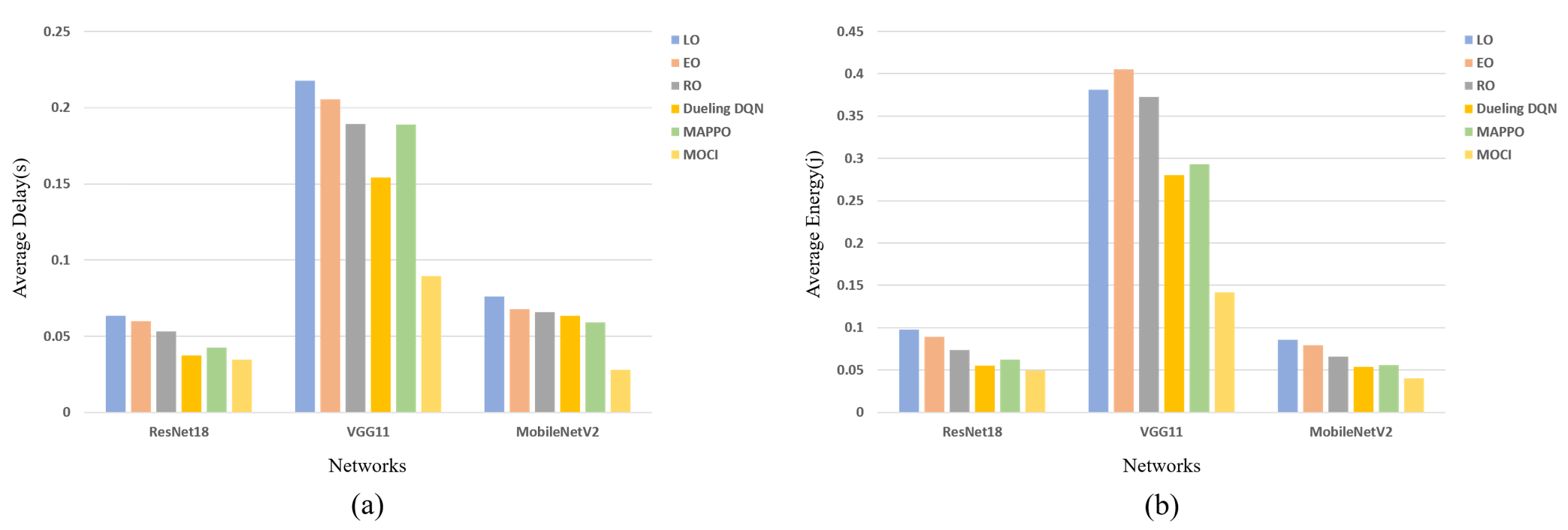


Figure S3: Comparison of different strategies with different DNN types

(a) Average delay of each algorithm on different networks. (b) Average energy consumption of each algorithm on different networks.


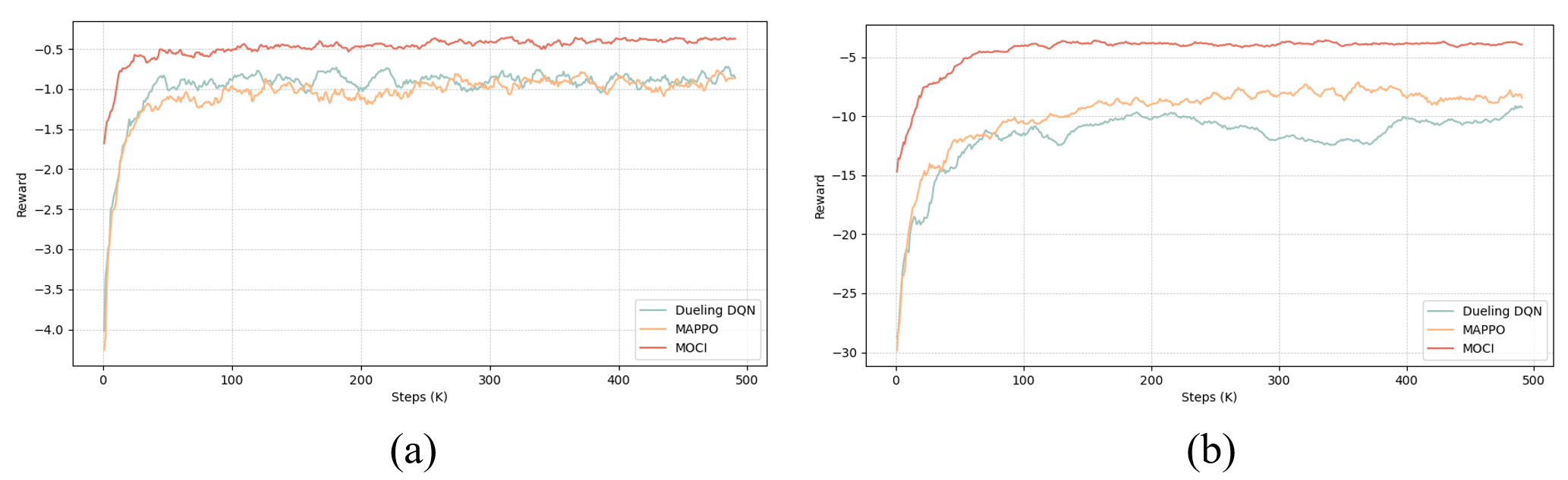


Figure S4: Convergence process of three strategies with different DNNs

(a) Convergence process of the three strategies under MobileNetV2. (b) Convergence process of the three strategies under VGG11.


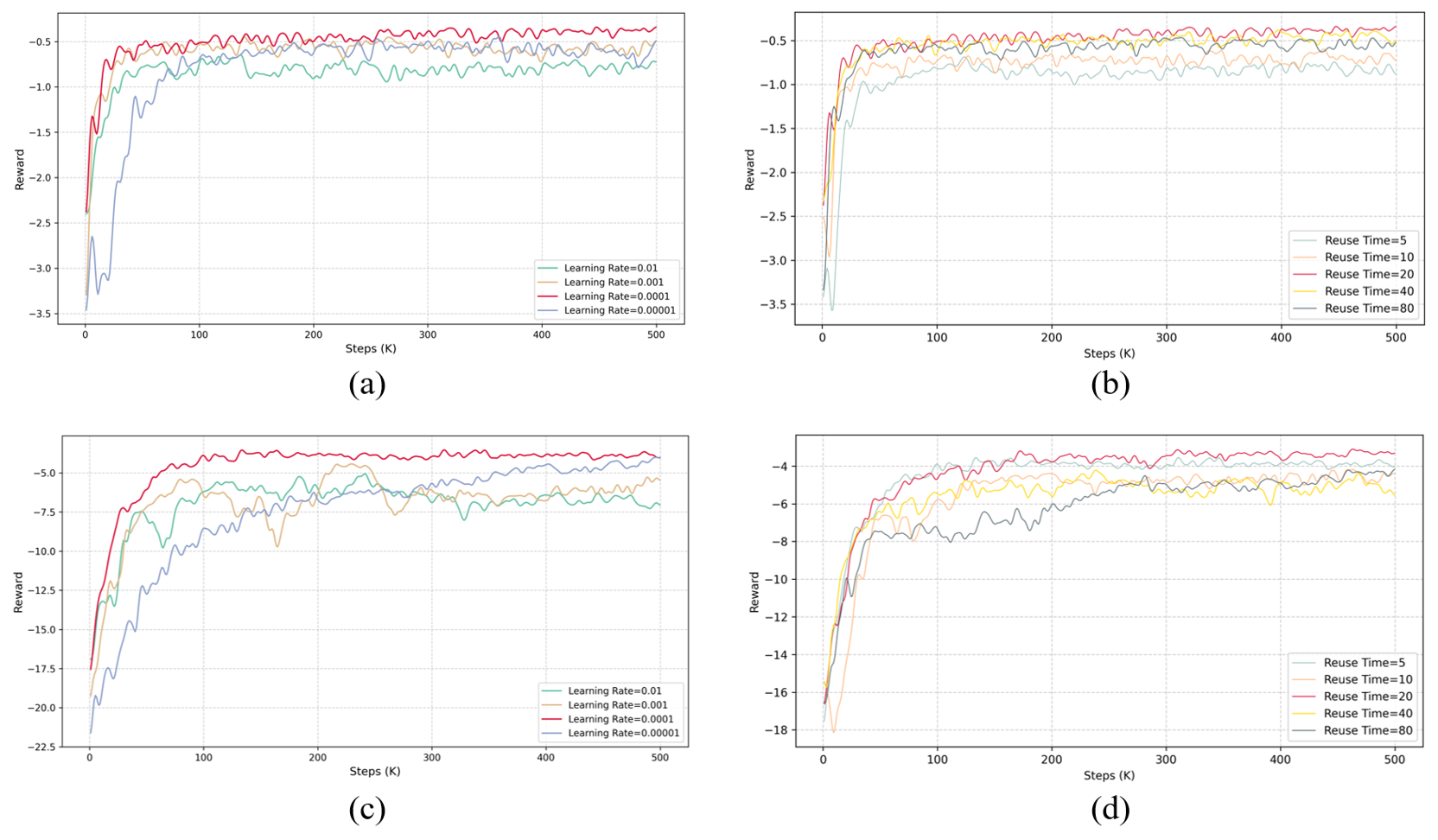


Figure S5: Convergence performance of different DNN architectures with different hyperparameter settings

(a) Convergence process of MobileNetV2 with different learning rates. (b) Convergence process of MobileNetV2 with different reuse times. (c) Convergence process of VGG11 with different learning rates. (d) Convergence process of VGG11 with different reuse times.

Table S1 Compression rate analysis of three models

| Model | Average compression rate (quantization) | | Compression rate analysis |
| --- | --- | --- | --- |
| ResNet18 | 50%-60% | Deeper networks and residual connections | |
| VGG11 | 70%-80% | Simple structure, many redundant parameters and easy to compress | |
| MobileNetV2 | 40%-50% | Highly optimized, less computational and parametric quantities | |

Table S2 Analysis of the effect of Wasserstein distance on strategy variability and performance stability

| Step(K) | Differences in action selection(%) | | Reward | |
| --- | --- | --- | --- | --- |
|  | unconstrained | constrained | unconstrained | constrained |
| 10 | 36 | 24 | -7.06 | -3.75 |
| 50 | 32 | 20 | -2.98 | -1.70 |
| 100 | 25 | 15 | -2.07 | -1.25 |
| 200 | 18 | 11 | -1.86 | -0.94 |
| 500 | 9 | 4 | -1.67 | -0.63 |

Table S3 Analysis of the optimization effect of LSTM and Wasserstein distance on delay, energy consumption and reward

| Module | Average delay(s) | Average energy(j) | Reward |
| --- | --- | --- | --- |
| No LSTM | 0.0373 | 0.0521 | -1.59 |
| LSTM | 0.0329 | 0.0462 | -1.05 |
| LSTM+Wasserstein | 0.0278 | 0.0392 | -0.43 |
